# Endobronchial ultrasound-guided transbronchial needle aspiration for lung cancer staging: early experience in Brazil*,**

**DOI:** 10.1590/S1806-37132015000100004

**Published:** 2015

**Authors:** Viviane Rossi Figueiredo, Paulo Francisco Guerreiro Cardoso, Márcia Jacomelli, Sérgio Eduardo Demarzo, Addy Lidvina Mejia Palomino, Ascédio José Rodrigues, Ricardo Mingarini Terra, Paulo Manoel Pego-Fernandes, Carlos Roberto Ribeiro Carvalho

**Affiliations:** University of São Paulo, School of Medicine, Hospital das Clínicas, São Paulo, Brazil. Heart Institute of the University of São Paulo School of Medicine Hospital das Clínicas, São Paulo, Brazil; University of São Paulo, School of Medicine, Hospital das Clínicas, São Paulo, Brazil. University of São Paulo School of Medicine Hospital das Clínicas, São Paulo, Brazil; University of São Paulo, School of Medicine, Hospital das Clínicas, São Paulo, Brazil. Heart Institute of the University of São Paulo School of Medicine Hospital das Clínicas, São Paulo, Brazil; University of São Paulo, School of Medicine, Hospital das Clínicas, São Paulo, Brazil. Bronchoscopy Department. Heart Institute of the University of São Paulo School of Medicine Hospital das Clínicas, São Paulo, Brazil; University of São Paulo, School of Medicine, Hospital das Clínicas, São Paulo, Brazil. Bronchoscopy Department. Heart Institute of the University of São Paulo School of Medicine Hospital das Clínicas, São Paulo, Brazil; University of São Paulo, School of Medicine, Hospital das Clínicas, São Paulo, Brazil. Bronchoscopy Department. Heart Institute of the University of São Paulo School of Medicine Hospital das Clínicas, São Paulo, Brazil; University of São Paulo, School of Medicine, Department of Cardiorespiratory Diseases, São Paulo, Brazil. Department of Cardiorespiratory Diseases, University of São Paulo School of Medicine, São Paulo, Brazil; University of São Paulo, School of Medicine, Department of Cardiorespiratory Diseases, São Paulo, Brazil. Thoracic Surgery Section, Department of Cardiorespiratory Diseases, University of São Paulo School of Medicine, São Paulo, Brazil; University of São Paulo, School of Medicine, Department of Cardiorespiratory Diseases, São Paulo, Brazil. Pulmonology Section, Department of Cardiorespiratory Diseases, University of São Paulo School of Medicine, São Paulo, Brazil

**Keywords:** Lung neoplasms, Neoplasm staging, Bronchoscopy, Endoscopic ultrasound-guided fine needle aspiration, Lymph nodes

## Abstract

**Objective:**

Endobronchial ultrasound-guided transbronchial needle aspiration (EBUS-TBNA) is a minimally invasive, safe and accurate method for collecting samples from mediastinal and hilar lymph nodes. This study focused on the initial results obtained with EBUS-TBNA for lung cancer and lymph node staging at three teaching hospitals in Brazil.

**Methods:**

This was a retrospective analysis of patients diagnosed with lung cancer and submitted to EBUS-TBNA for mediastinal lymph node staging. The EBUS-TBNA procedures, which involved the use of an EBUS scope, an ultrasound processor, and a compatible, disposable 22 G needle, were performed while the patients were under general anesthesia.

**Results:**

Between January of 2011 and January of 2014, 149 patients underwent EBUS-TBNA for lymph node staging. The mean age was 66 ± 12 years, and 58% were male. A total of 407 lymph nodes were sampled by EBUS-TBNA. The most common types of lung neoplasm were adenocarcinoma (in 67%) and squamous cell carcinoma (in 24%). For lung cancer staging, EBUS-TBNA was found to have a sensitivity of 96%, a specificity of 100%, and a negative predictive value of 85%.

**Conclusions:**

We found EBUS-TBNA to be a safe and accurate method for lymph node staging in lung cancer patients.

## Abstract (pt)

**Objetivo:**

A *endobronchial ultrasound-guided transbronchial needle aspiration* (EBUS-TBNA, punção aspirativa por agulha guiada por ultrassom endobrônquico) é um método seguro e preciso para a coleta de amostras de linfonodos mediastinais e hilares. O presente estudo teve por objetivo avaliar os resultados iniciais com EBUS-TBNA para o estadiamento linfonodal de câncer de pulmão em 3 hospitais acadêmicos no Brasil.

**Métodos:**

Análise retrospectiva de pacientes com neoplasia de pulmão diagnosticada e submetidos a EBUS-TBNA para estadiamento linfonodal mediastinal. Todos os procedimentos foram realizados sob anestesia geral. Utilizou-se um ecobroncoscópio, uma processadora de ultrassom e agulhas 22 G descartáveis e compatíveis com o ecobroncoscópio.

**Resultados:**

Entre janeiro de 2011 e janeiro de 2014, 149 pacientes foram submetidos ao estadiamento linfonodal com EBUS-TBNA. A média de idade foi 66 ± 12 anos, sendo 58% do sexo masculino. Um total de 407 linfonodos foram puncionados via EBUS-TBNA. Os tipos mais comuns de neoplasia brônquica foram adenocarcinoma (em 67%) e carcinoma escamoso (em 24%). Para o estadiamento da neoplasia, o EBUS-TBNA apresentou sensibilidade de 96%, especificidade de 100% e valor preditivo negativo de 85%.

**Conclusões:**

A EBUS-TBNA mostrou-se um método seguro e acurado no estadiamento linfonodal em pacientes com câncer de pulmão.

## Introduction

Lung cancer is the leading cause of cancer death, having accounted for an estimated 160,000 deaths in the United States in 2012.^(^ 1 ^)^ In Brazil, there are approximately 27,000 new cases of lung cancer every year.^(^ 2 ^)^ The prognosis depends on early diagnosis, histology, and staging. The acquisition of CT scans and positron emission tomography/CT (PET/CT) scans represent important steps in the lung cancer staging process. Mediastinal lymph node sampling for cytology and histopathology is essential for accurate staging, because it provides the guidelines for treatment and can avoid unnecessary surgery. Samples for lymph node cytology and histopathology can be obtained endoscopically (by conventional or ultrasound-guided bronchoscopic needle aspiration biopsy) or surgically (by mediastinoscopy, mediastinotomy, mediastinal sampling, or mediastinal lymph node dissection). 

Endobronchial ultrasound-guided transbronchial needle aspiration (EBUS-TBNA), a minimally invasive method, has been shown to be safe and accurate for collecting samples from mediastinal and hilar lymph nodes. In mediastinal lymph node staging for lung cancer, EBUS-TBNA has been shown to have an accuracy of 98%^(^ 3 ^)^ and has proven to be superior to CT and PET/CT, even in the absence of mediastinal adenopathy (defined as a lymph node diameter ≥ 10 mm) on the CT scan. Because EBUS-TBNA is a nonsurgical procedure, it causes less discomfort to the patient and can be performed on an outpatient basis, resulting in treatment costs that are lower than those associated with mediastinoscopy.^(^ 4 ^)^

Prospective studies comparing EBUS-TBNA and mediastinoscopy have shown that, in the lymph node staging of non-small cell lung cancer, there is a high level of concordance between the two methods.^(4, 5)^ In addition, EBUS-TBNA has the ability to identify contralateral hilar lymph node metastases. This examination begins with the N3 lymph nodes, progressing towards the N2 and N1 lymph node stations. Sampling includes any lymph node station with nodes greater than 5 mm in diameter on their short axis.

Implementing EBUS-TBNA in Brazil has required planning; training of medical and nursing staff; changes to the physical structure of facilities, a new operational protocol; and a billing strategy suited to use of the procedure in Brazil.^(^ 6 ^)^ This study focused on the results obtained with EBUS-TBNA for lung cancer staging at three teaching hospitals in Brazil.

## Methods

This was a retrospective cross-sectional study that included patients ≥ 18 years of age diagnosed with lung cancer who underwent EBUS-TBNA for lymph node staging between January of 2011 and January of 2014. The study was approved by the Research Ethics Committee of the University of São Paulo School of Medicine *Hospital das Clínicas*, in the city of São Paulo, Brazil (Protocol no. 435.645).

Every EBUS-TBNA procedure was performed by one of three experienced bronchoscopists, all of whom had been trained in standard and interventional bronchoscopy, with similar levels of training in EBUS-TBNA, each having performed the procedure in more than 50 cases. In all instances, endobronchial ultrasound scope was used (BF-UC180F; Olympus Medical Systems, Tokyo, Japan). Ultrasound imaging was generated by one of two different ultrasound processors-EU-ME1 (Olympus Medical Systems); or Prosound α5 (Aloka, Tokyo, Japan)-and we used a disposable 22 G needle compatible with the ultrasound scope-NA-201SX-4022 (Olympus Medical Systems); ECHO-HD-22-EBUS-O (Cook Medical, Winston-Salem, NC, USA); or GUS-45-18-022 (Medi-Globe, Achenmühle, Germany). The procedure was performed either in the operating room or in the endoscopy suite. In most of the procedures, a trained cytopathologist was present to analyze the lymph node cytological aspirates and determine whether the material collected was satisfactory for diagnosis. The lymph node map reported by Yasufuku et al.^(^ 7 ^)^ was used in order to locate the lymph node stations in all procedures.

As a means of checking the airways for endoluminal lesions, the EBUS-TBNA was preceded by conventional bronchoscopy under local anesthesia (1% xylocaine instilled into the airway). If any such lesions were identified, biopsies were taken only at completion of the EBUS-TBNA staging. The EBUS-TBNA followed standardized steps, from the assessment of CT and PET/CT images to the procedure itself. Planning for the procedure included determining which lymph node stations would be sampled in a sequence of aspiration biopsies, performed initially at the lymph node station farthest from the tumor (N3 station), then moving on to the lymph node stations closer to the tumor (N2 and N1).^(^ 8 ^)^ The procedure was performed with the patient under general anesthesia, the airway being maintained with a laryngeal mask or endotracheal intubation. Prior to sampling, the mediastinal lymph node stations were mapped, measured, and photographed. The lymph node contents were aspirated in order to collect cell aspirates for cytology, and tissue samples for histology were also collected. The collected material was pushed out from the needle tract by the guidewire onto glass slides or into a container. A single drop of the material was placed on the glass slide and a uniform smear was then produced. The remaining material within the needle tract was flushed out into 10% formaldehyde solution for cell-block preparation. Solid samples were placed into a separate container with 10% formaldehyde solution for histopathology. In the pathology department, fragments and sediment were embedded in paraffin for histology and immunohistochemistry.

The data collected are presented as absolute numbers and percentages. Sensitivity, specificity and negative predictive value for the detection of lymph node metastasis were calculated as follows: 

sensitivity = *tp */ (*tp+fn*)

specificity = *tn */ (*tn+fp*)

positive predictive value = *tp */ (*tp+fp*)

negative predictive value = *tn */ (*tn+fn*)

where *tp* is true positive; *tn* is true negative; *fp* is false positive; and *fn* is false negative. The size difference between malignant and benign lymph nodes was calculated using a z test. The level of significance was set at p < 0.05.

## Results

**Figure 1 -:**
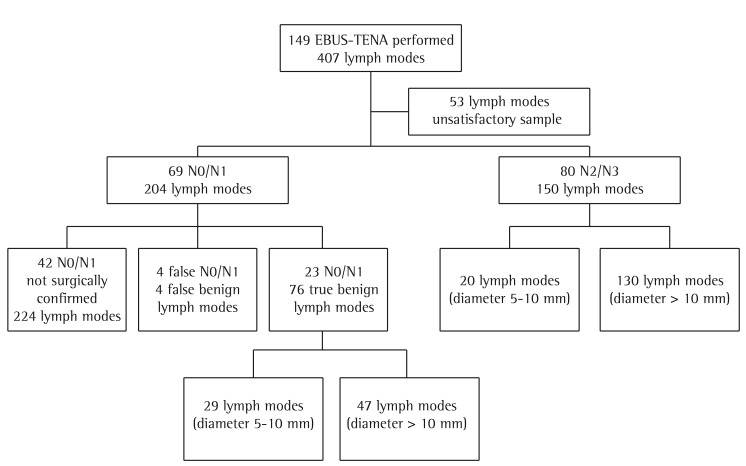
Flow diagram of patient enrollment, lymph nodes sampled, and endobronchial ultrasound-guided transbronchial needle aspiration (EBUS-TBNA) results.

One hundred and forty-nine patients diagnosed with lung cancer underwent EBUS-TBNA for lymph node staging. The mean age was 66 ± 12 years, and 87 (58%) of the patients were male. Figure 1 summarizes patient enrollment and the results of the EBUS-TBNA. On the basis of the EBUS-TBNA findings, the cancer was staged as N0/N1 in 69 patients (46%) and as N2/N3 in 80 (54%). The histopathology and mediastinal lymph node staging by EBUS-TBNA are described in Table 1. The tumor histology showed that, in our sample, the most common type of neoplasia was adenocarcinoma, which was identified in 100 cases (67%), followed by squamous cell carcinoma, in 36 (24%), small cell carcinoma, in 7 (5%), carcinoid tumor, in 3 (2%), sarcoma, in 2 (1%) and mucoepidermoid carcinoma, in 1 (0.5%). The EBUS-TBNA identified lung cancer and N0/N1 lymph nodes in 69 patients, of whom 27 (39%) underwent surgical lymph node staging. There were 23 true-positive results and 4 false-negative results (3 adenocarcinomas and 1 carcinoid tumor). Of the remaining 42 patients (those in whom the staging was not confirmed by surgery), 32% were considered unfit for surgery, 17% had distant metastases, 4% had another primary tumor outside the lung, and 13% were lost to follow up. There was one procedure-related complication-endobronchial bleeding at the puncture site in a patient with small cell carcinoma-which was controlled endoscopically. There was no procedure-related mortality among the patients in our sample. 

**Table 1 -:** Histopathology and mediastinal lymph node staging by endobronchial ultrasound-guided transbronchial needle aspiration.

Histopathology	Nº of patients (%)			
	Total	N0/N1	N2	N3
Adenocarcinoma	100 (67)	46 (46)	34 (34)	20 (20)
Squamous cell carcinoma	36 (24)	17 (47)	14 (39)	5 (14)
Small cell carcinoma	7 (5)	1 (14)	5 (72)	1 (14)
Carcinoid tumor	3 (2)	2 (67)	0	1 (33)
Sarcoma	2 (1)	2 (100)	0	0
Mucoepidermoid carcinoma	1 (0,7)	1 (100)	0	0
Total	149 (100)	69 (46)	53 (36)	27 (18)

**Figure 2 -:**
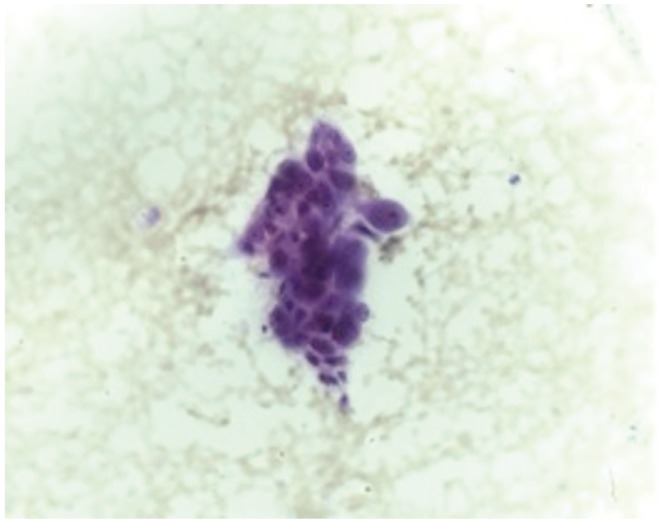
Fine needle aspiration cytology of a lymph node with squamous carcinoma (thionin staining; magnification, ×400). Courtesy of the Pathology Laboratory, Heart Institute, University of São Paulo School of Medicine Hospital das Clínicas, São Paulo, Brazil.

**Figure 3 -:**
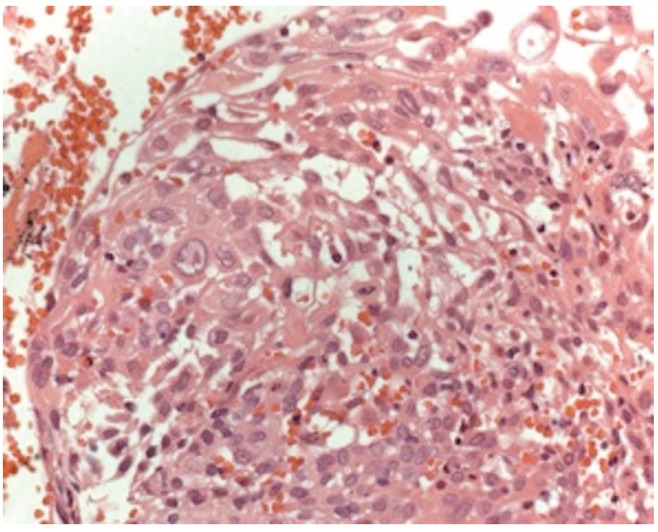
Cell block histopathology of a lymph node with squamous carcinoma (Hematoxylin-eosin staining; magnification, ×400). Courtesy of the Pathology Laboratory, Heart Institute, University of São Paulo School of Medicine Hospital das Clínicas, São Paulo, Brazil.

A total of 407 lymph nodes with a diameter ≥ 5 mm were assessed by EBUS-TBNA, with an average of 3.15 lymph nodes per patient. Fifty-three (13%) of the punctures were considered unsatisfactory for analysis by the pathologist. The lymph node stations sampled were 2R (0.8%); 2L (0.4%); 4R (23.5%); 4L (14.0%); 7 (30.8%); 10R (6.0%); 10L (4.6%); 11R (8.8%); 11L (10.4%); 12R (0.4%); and 12L (0.2%). Cytology and histology samples are depicted in Figures 2 and 3, respectively.

The final cytologic diagnosis and diameter of lymph nodes are described in Table 2. As far as lymph node size is concerned, the comparison between benign and malignant lymph nodes showed that those > 10 mm in diameter were more often malignant. Of the 407 lymph nodes biopsied, 76 (19%) were classified as benign on the basis of the surgical findings. Of those 76 lymph nodes, 47 (62%) had a diameter > 10 mm and 29 (38%) had a diameter of 5-10 mm, reactive lymphadenitis being identified in 75 and one testing positive for tuberculosis. The finding of malignancy in lymph nodes that had been classified as unsuspicious by other imaging methods (PET/CT and CT scan) resulted in a change in the management strategy in 5 patients (corresponding to 33% of the patients in whom PET/CT and CT scans had raised no suspicion about the mediastinal lymph nodes in question). Lymph node diameters and final cytologic diagnoses are shown in Table 2. For the detection of lymph node metastasis in our sample, EBUS-TBNA had a sensitivity of 96% (103 of 117), a specificity of 100%, and a negative predictive value of 85% (23 of 27).

**Table 2 -:** Final cytologic diagnosis in 407 lymph nodes.

Final cytologic diagnosis	Nº of lymph nodes (%)		
	n (%)	Diameter	
		5-10 mm	> 10 mm
Positive for malignant cells	150 (37)	20 (13)	130 (87)*
Benign^a^	76 (19)	29 (38)	47 (62)*
Negative for malignant cells^b^	128 (31)	38 (30)	90 (70)
False benign	4 (1)	1 (25)	3 (75)
Unsatisfactory sample	53 (13)	7 (13)	46 (87)
Total	407	91 (22)	316 (78)

* significant difference (p < 0.001; 95% CI: 0.137-0.363).

a Confirmed by surgery.

b Not confirmed by surgery.

## Discussion

Preliminary results from the first three centers in Brazil at which it was implemented have demonstrated that the EBUS-TBNA procedure is a safe and accurate method for staging lung cancer. The preference for general anesthesia over conscious sedation is based on the comfort the former provides the patient as well as the EBUS-TBNA team. Performing the cytopathology in the room often adds time to the procedure in exchange for more accurate results. However, the routine use of general anesthesia increases costs and procedural time (in the operating room or endoscopy suite). As far as accuracy is concerned, one study comparing deep and conscious sedation for EBUS-TBNA cancer staging showed that more lymph nodes were sampled under deep sedation than under conscious sedation, the diagnostic yields being 80% and 66%, respectively.^(^ 9 ^)^ Because the EBUS-TBNA procedure is not only technically demanding but also labor-intensive, we believe that either deep sedation or general anesthesia are necessary in order to perform the procedure easily and safely. In addition, instruction in EBUS-TBNA at teaching hospitals is facilitated if the procedure is performed under general anesthesia. At some facilities, EBUS-TBNA is performed with a rigid bronchoscope under general anesthesia.^(^ 10 ^)^ At others, it is performed through an endotracheal tube under deep sedation or through a laryngeal mask under general anesthesia.^(11, 12)^ At our facility, the preference is for performing the procedure through a laryngeal mask under general anesthesia. 

Various strategies for approaching mediastinal and hilar lymph nodes have been proposed. ^(^ 8 ^)^ Lymph node puncture can be performed concurrently with the lymph node mapping, the lymph nodes with malignant characteristics, such as diameter > 10 mm, spherical shape, well-defined margins, necrosis, heterogeneity, and absence of hilum, being identified selectively.^(^ 13 ^)^ In our study, we elected to do the ultrasound mapping of all accessible lymph nodes prior to the puncture of the lymph node stations. As recommended by other authors,^(11,^ ^13)^ we sampled lymph nodes ≥ 5 mm in diameter, which would presumably have a greater impact on staging and management.

We found that, among the lung cancer patients evaluated in the present study, lymph nodes > 10 mm in diameter were more often malignant, as reported in the literature. However, Herth et al.^(^ 14 ^)^ reported that, for the detection of metastatic lymph nodes of 5-10 mm in diameter, EBUS-TBNA has a sensitivity of 89% and a negative predictive value of 98.9%. In our study, 13% of the lymph nodes positive for malignancy were ≤ 10 mm in diameter. That finding resulted in a change in the management of the cancer in 5 patients (33% of the patients in whom mediastinal lymph nodes had been classified as unsuspicious on PET/CT and CT scans). In this context, EBUS-TBNA might play an important role in the approach to mediastinal and hilar lymph nodes initially considered metastatic or nonmetastatic solely on the basis of their diameter, as determined by other imaging methods. Our data show that 62% of the truly benign mediastinal lymph nodes were > 10 mm in diameter, whereas 13% of the true malignant mediastinal lymph nodes were smaller than 10 mm in diameter. Another study comparing CT, PET/CT, and EBUS-TBNA for the detection of lymph node metastasis in a sample of 102 patients with lung cancer showed that the accuracy of CT, PET/CT, and EBUS-TBNA was 60.8%, 72.5%, and 98%, respectively.^(^ 3 ^)^

In EBUS-TBNA samples, the lymph node morphology can also suggest malignancy. Certain characteristics, such as being round, having heterogeneous density, showing necrosis, and having well-defined margins can be suggestive of malignancy. Conversely, having a diameter < 10 mm, having an oval shape, having homogeneous density, showing no necrosis, having ill-defined margins and the presence of a central hilar structure are suggestive of a benign lymph node.^(^ 13 ^)^

The present study has certain limitation. Because of the retrospective design and the small number of patients, we cannot draw any correlations between lymph node ultrasound patterns and the presence of metastasis. Nevertheless, given the high prevalence of infectious granulomatous diseases in our patient population, the recognition and differentiation of lymph node ultrasound patterns might become important for discerning between benign and malignant disease. 

We consider the presence of a cytopathologist during the procedure essential to determining whether the material collected is satisfactory (in terms of volume and character) for diagnosis. A cytopathologist can also contribute to the screening and processing of samples for other procedures, such as histochemistry, immunohistochemistry, genetic mutation testing, and culture.^(^ 15 ^)^

It has been reported that the learning curve for EBUS-TBNA is "steep", the performance of at least 50 procedures, under the supervision of an experienced bronchoscopist, being required in order to train a specialist.^(^ 7 ^)^ In fact, in a preliminary study involving only 50 patients, we found that only 74% of the samples were considered satisfactory for cytology.^(^ 16 ^)^ Since then, that proportion has increased considerably as we have gained expertise and started using on-site cytopathology during the EBUS-TBNA procedures. Other authors have questioned whether it is necessary to have a cytopathologist in the room in order to improve the diagnostic accuracy of EBUS-TBNA.^(^ 17 ^)^ After our initial experience with EBUS-TBNA was reported, improvements were made in the technique, as well as in the collection, preparation, and processing of samples, and team experience was enhanced. Most importantly, the presence of a cytopathologist in the room has reduced the proportion of samples considered unsatisfactory for diagnosis from 26% in the first year of our experience to 13% at this writing. 

There is considerable evidence that EBUS-TBNA is a safe procedure, the reported rate of complications-including minor complications such as bronchospasm and endobronchial bleeding, as well as more severe complications, such as pneumomediastinum and mediastinitis-ranging from 0.5% to 1.2%.^(^ 18 ^)^ In the present study, we observed only one procedure-related complication among the 149 patients evaluated, and there were no procedure-related deaths. 

New methods aimed at improving EBUS-guided sample collection have been reported.^(^ 19 ^)^ Such methods include the use of mini-forceps for the collection of lymph node fragments through small perforations on the bronchial wall.^(^ 19 ^)^ In addition, analysis of the genetic profile of the tumors in the samples collected has recently been included in EBUS-TBNA protocols.^(20, 21)^ Within this context, changes in the procedure, as well as in the collection and analysis of the samples, are indicators of the ongoing technological development of EBUS-TBNA. 

In conclusion, the early results obtained with EBUS-TBNA in Brazil indicate that it is a safe and accurate procedure for lung cancer staging. It is a minimally invasive procedure whose results can have a significant impact on the therapeutic strategy, the chance of a patient undergoing unnecessary surgery being higher when treatment decisions are based on radiological findings alone. The success of EBUS-TBNA depends on collaboration among the members of a multidisciplinary team composed of medical (bronchoscopist, cytopathologist and anesthesiologist) and paramedical staff.
